# Neuroendocrine tumor secondary to pulmonary hypoplasia: A case report

**DOI:** 10.1111/1759-7714.14374

**Published:** 2022-03-17

**Authors:** Takuro Yukawa, Yuta Ishida, Yoshio Naomoto, Yasumasa Monobe, Takuya Fukazawa, Tomoki Yamatsuji

**Affiliations:** ^1^ Department of General Surgery Kawasaki Medical School Okayama Japan; ^2^ Department of Pathology 1 Kawasaki Medical School Okayama Japan

**Keywords:** carcinoid, neuroendocrine cell hyperplasia, pulmonary hypoplasia, tumorlet

## Abstract

Pulmonary hypoplasia is diagnosed during the perinatal period and is a cause of death in newborns. However, these developmental abnormalities are diagnosed in adulthood in some cases. A 70‐year‐old male smoker was diagnosed with stage IIIA pulmonary adenocarcinoma in the right upper lobe with right middle lobe hypoplasia. He subsequently underwent right upper and middle lobectomy with lymph node dissection by video‐assisted thoracoscopic surgery. In addition to an invasive adenocarcinoma in the right upper lobe, pathological examination of the hypoplastic lobe revealed neuroendocrine hyperplasia, as well as tumorlets and a typical carcinoid. Eight cases of pulmonary neuroendocrine tumors that developed from pulmonary hypoplasia have been reported to date. Interestingly, all but one case occurred in the right middle lobe. Neuroendocrine cell hyperplasia has been reported to develop in hypoplastic lungs postnatally; therefore, we speculated that the lesion was the origin of these neuroendocrine tumors. Moreover, the pathological findings suggested that atelectasis was involved in the pathogenesis of this rare condition. In adults, when lobar hypoplasia is diagnosed, neuroendocrine tumors should be anticipated.

## Graphical abstract

Here, we report the case of a patient who was diagnosed with pulmonary adenocarcinoma in the right upper lobe and middle lobe hypoplasia; the patient underwent right upper and middle lobectomy with lymph node dissection. In addition to adenocarcinoma in the upper lobe, pathological examination of the hypoplastic lobe revealed neuroendocrine hyperplasia, as well as tumorlets and a typical carcinoid. In adults, when lobar hypoplasia is diagnosed, neuroendocrine tumors should be anticipated.

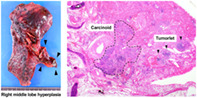

## INTRODUCTION

Pulmonary hypoplasia is a rare disease characterized by incomplete development of the lungs. It is a known cause of death in newborns^1^, ^2^; however, it can be diagnosed in adulthood in some cases. Most pulmonary hypoplasia are secondary to an underlying abnormality, such as congenital diaphragmatic hernia.^3^ In addition, it has been reported that neuroendocrine cell hyperplasia originates from pulmonary hypoplasia.^4^ Here, we report the case of a 70‐year‐old patient with hypoplasia of the right middle lobe medial segment complicated by a peripheral carcinoid and tumorlets.^5^, ^6^

## CASE REPORT

**FIGURE 1:**
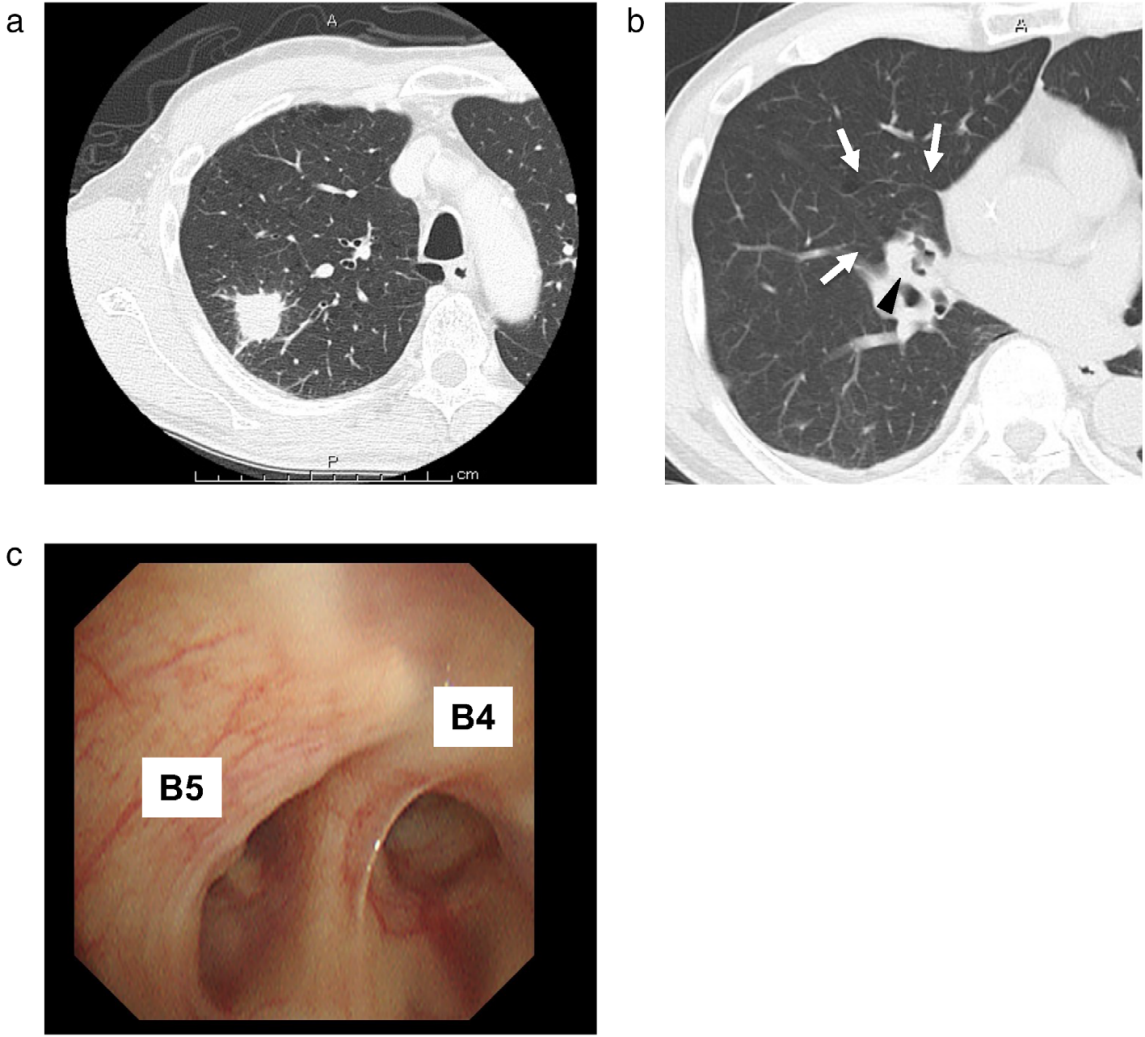
(a) Contrast‐enhanced computed tomography (CE‐CT) showing a 32 mm irregular nodule in the posterior segment (S2) of the upper lobe of the right lung. (b) A small‐sized right middle lobe is detected on computed tomography (white arrowhead). The arrowhead indicates the atelectatic region. (c) Bronchoscopy revealing no abnormal findings in the bifurcation of the right middle lobe bronchus (B4 and B5)

A 70‐year‐old male smoker (48 pack‐years) was referred to our hospital after being diagnosed with arteriosclerosis obliterans. In addition to stenosis of the left and right external iliac artery, contrast‐enhanced computed tomography (CE‐CT) revealed an irregular nodule in the upper lobe of the right lung (Figure 1a). The volume of the middle lobe was much smaller than that of the other lobes (Figure 1b); however, bronchoscopy showed no abnormal findings in the orifices of the segmental bronchi (Figure 1c). Transbronchial lung biopsy revealed pulmonary adenocarcinoma, and the patient underwent lobectomy with lymph node dissection for stage 1A3 (cT1cN0M0) lung cancer.

Intraoperatively, the small middle lobe of the right lung was considered hypoplastic as pulmonary veins and undeveloped arteries were observed. In addition to an incomplete fissure between the upper and middle lobes, lung hypoplasia can cause pneumonia. Therefore, the right upper and middle lobes and lymph nodes at the 2R, 4R, and 10 levels were resected.

**FIGURE 2:**
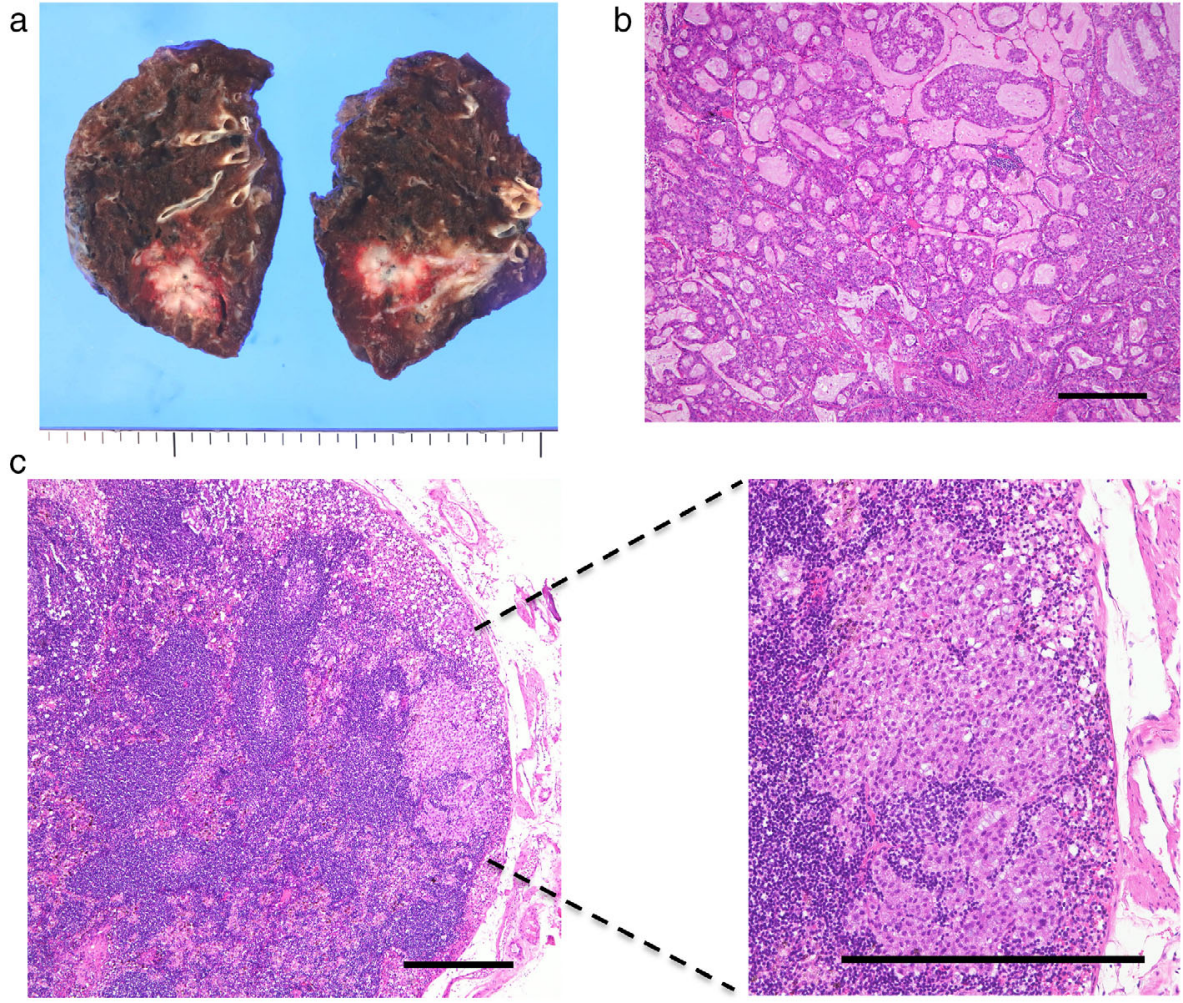
(a) Macroscopic findings showing a 4.0 cm diameter tumor in the upper lobe of the right lung. (b) Macroscopic findings of the tumor. The pathological diagnosis is a moderately invasive adenocarcinoma with acinar predominantly showing a mucinous cribriform pattern. Scale bar, 500 μm. (c) Metastasis in a right lower paratracheal node (station 4R). Scale bar, 500 μm

**FIGURE 3:**
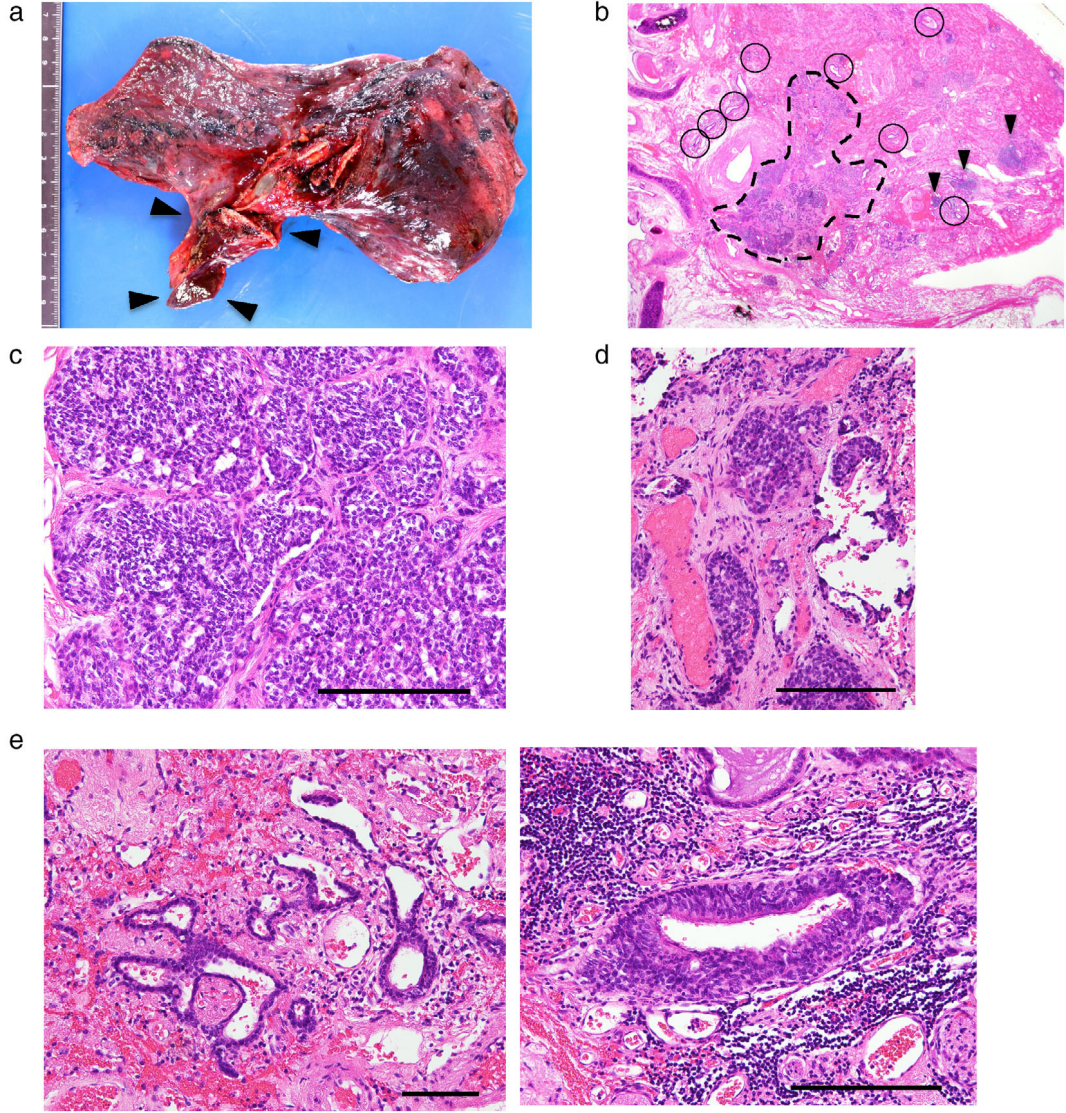
(a) Macroscopic findings in the right upper and lower lobes. The middle lobe (black arrows, 6 cm) is much smaller than the upper lobe and is diagnosed as hypoplasia. (b) Neuroendocrine cell hyperplasia (circled), tumorlets (arrowheads), and a typical carcinoid (delineated by broken line) are observed in a Loupe view using hematoxylin–eosin (HE) staining. (c) Microscopic findings of the carcinoid. The tumor has a monotonous small ovoid chromatin pattern without mitosis. Little necrosis is observed. Scale bar, 200 μm. (d) Pathological findings of the tumorlets. The lesions are less than 5 mm. Scale bar, 200 μm. (e) Microscopic findings of the neuroendocrine cell hyperplasia. The foci are limited to the bronchiole. Scale bar, 200 μm

The lung cancer in S2 was an invasive adenocarcinoma (Figure 2a,b). In addition, intrapulmonary metastasis in the same lobe (pT3, pm1) and hilar and mediastinal lymph node metastases were observed (pN2), and the pathological stage was stage IIIA (Figure 2c). The middle lobe was pathologically confirmed to be hypoplastic (Figure 3a). Of note, pulmonary neuroendocrine cell hyperplasia, as well as multiple tumorlets and a typical carcinoid, were observed in the right middle lobe (Figures 3b–e and S1). Given the patient's poor performance status postoperatively, he has been followed‐up without any adjuvant treatment.

## DISCUSSION

In this report, we describe a rare case of right middle lobe hypoplasia associated with tumorlets and a typical carcinoid. Most pulmonary hypoplasia cases are secondary to an underlying abnormality, such as chest congenital diaphragmatic hernia, chest wall malformations, oligohydramnios, and neuromuscular disorders that interfere with fetal respiration.^3^ The patient had none of the pre‐existing conditions described above, suggesting that the hypoplasia was primarily caused by an embryological lung defect.^7^ The patient had no previous history of bronchitis or pneumonia. While lung agenesis and aplasia are usually diagnosed soon after birth due to their early clinical presentation, lobar hypoplasia can be asymptomatic for a long time.

To date, nine cases of pulmonary hypoplasia associated with pulmonary neuroendocrine tumors, including our case, have been reported in the literature (Table 1).^8^, ^9^, ^10^, ^11^, ^12^ Interestingly, all but one of them involved the right middle lobe. However, the causal association between these tumors and hypoplasia is unknown. Hypoplasia of the lungs is one of the main causes of mortality in infants with congenital diaphragmatic hernia (CDH). In cases of CDH, hyperplasia of neuroendocrine cells is found in the hypoplastic ipsilateral lung, and the speculated mechanism is a compensatory increase in neuroendocrine cells associated with impaired lung growth.^13^ Although our patient had no history of CDH, the observations suggest that neuroendocrine hyperplasia occurred after birth due to activation of neuroendocrine cells to compensate for tissue remodeling of the hypoplastic lung. In addition, neuroendocrine cell hyperplasia, tumorlets, and a carcinoid developed in the atelectatic area of the right middle lobe (Figures 1b and 3b), and hypoxia might have promoted the hyperplasia in this region.^14^ From the pathogenesis of the middle lobe syndrome, the lobe is prone to atelectasis. Therefore, it explains why most of the previously reported cases involved the middle lobe.

**TABLE 1:** Characteristics of reported cases of neuroendocrine tumors secondary to lobar hypoplasia in Japan, including our patient

Case no.	Age	Sex	Site of hypoplasia	Tumor size (mm)	Histology	Pathlogical stage	Associated abnormality	Smoking index	Author	Manuscript
1	61	Male	Right middle lobe	5	Typical carcinoid	IA1	(–)	1440	Yoshida et al.	Four patients with right middle lobe hypoplasia complicated by primary lung cancer. Jpn J Chest Surgy 32, 517‐522. 2018.
2	60	Female	Right middle lobe	10	Small cell lung cancer	IIB	(–)	0		
3	64	Male	Right middle lobe	5	Typical carcinoid	IA1	(–)	880		
4	68	Male	Right middle lobe	16	Typical carcinoid	IA2	(–)	900		
5	76	Female	Right middle lobe	8	Typical carcinoid	IA1	(–)	200	Sato et al.	A case of ACTH‐producing bronchopulmonary carcinoid tumor with Cushing's syndrome resected by video assisted thoracoscopic surgery. JJLC 41,161‐4. 2001.
6	68	Male	Right middle lobe	12	Typical carcinoid with tumorlet and DIPNECH	IA2	(–)	200	Maeshiro et al.	A case of diffuse idiopathic pulmonary neuroendocrine cell hyperplasia (DIPNECH) with typical carcinoid on lobar hypoplasia of middle lobe of lung. Jpn J Chest Surgy 30, 510‐24. 2016.
7	73	Female	Right lower lobe	less than 5	Tumorlet	NA	(–)	0	Yagyu et al.	A case of lung tumorlets secondary to pulmonary hypoplasia with recurrent haemoptysis. Respirol Case Rep. 6, e00373. 2018.
8	70	Male	Right middle lobe	30	Typical carcinoid	IA3	(–)	400	Motono et al.	Pulmonary Typical carcinoid on right middle lobe hypoplasia: A case report and review of the literature. Clin Image Case Rep J. 2, 120. 2020.
9	70	Male	Right middle lobe	5	Typical carcinoid with tumorlet and neurendocrine cell hyperplasia	IA1	(–)	960	Our case	

Abbreviations: ACTH, adrenocorticotropic hormone; DIPNECH, diffuse idiopathic pulmonary neuroendocrine cell hyperplasia; NA, not applicable.

Diffuse idiopathic pulmonary neuroendocrine cell hyperplasia (DIPNECH) is an idiopathic proliferation of pulmonary neuroendocrine cells and a known precursor of pulmonary neuroendocrine tumors.^15^ DIPNECH is more common in the lungs of non‐smoking women between the ages of 50 and 60 years without prior lesions. However, the neuroendocrine cell hyperplasia in our case occurred in the atelectatic region of the right middle lobe of a male smoker and was considered distinct from DIPNECH.

To date, nine cases of pulmonary neuroendocrine tumors that developed from pulmonary hypoplasia, including our case, have been reported. Interestingly, all the reported cases were from Japan (Table 1). We hope that this case report will contribute to the diagnosis of neuroendocrine tumors.

To conclude, we reported a case of tumorlets and a typical carcinoid secondary to right middle lobe hypoplasia. In adults, when lobar hypoplasia is diagnosed, neuroendocrine tumors should be anticipated and followed up.

## CONFLICT OF INTEREST

The authors report no competing interest.

## Supporting information


**Figure S1** (a) Immunohistochemistry showing diffuse synaptophysin staining in the carcinoid (left column). The KI‐67 index is less than 2% (right column). Scale bar, 1 mm. (b) Synaptophysin expression in a tumorlet. Scale bar, 200 μm. (c) Synaptophysin expression in diffuse idiopathic pulmonary neurorndocrine cell hyperplasia (left cloumn). Scale bar, 200 μm
