# Supplementary figures and images for: Neuroendocrine tumor secondary to pulmonary hypoplasia: A case report
Source: Thorac Cancer. 2022 Mar 17;13(8):1227–31. doi: 10.1111/1759-7714.14374 (PMC9013649; doi:10.1111/1759-7714.14374)

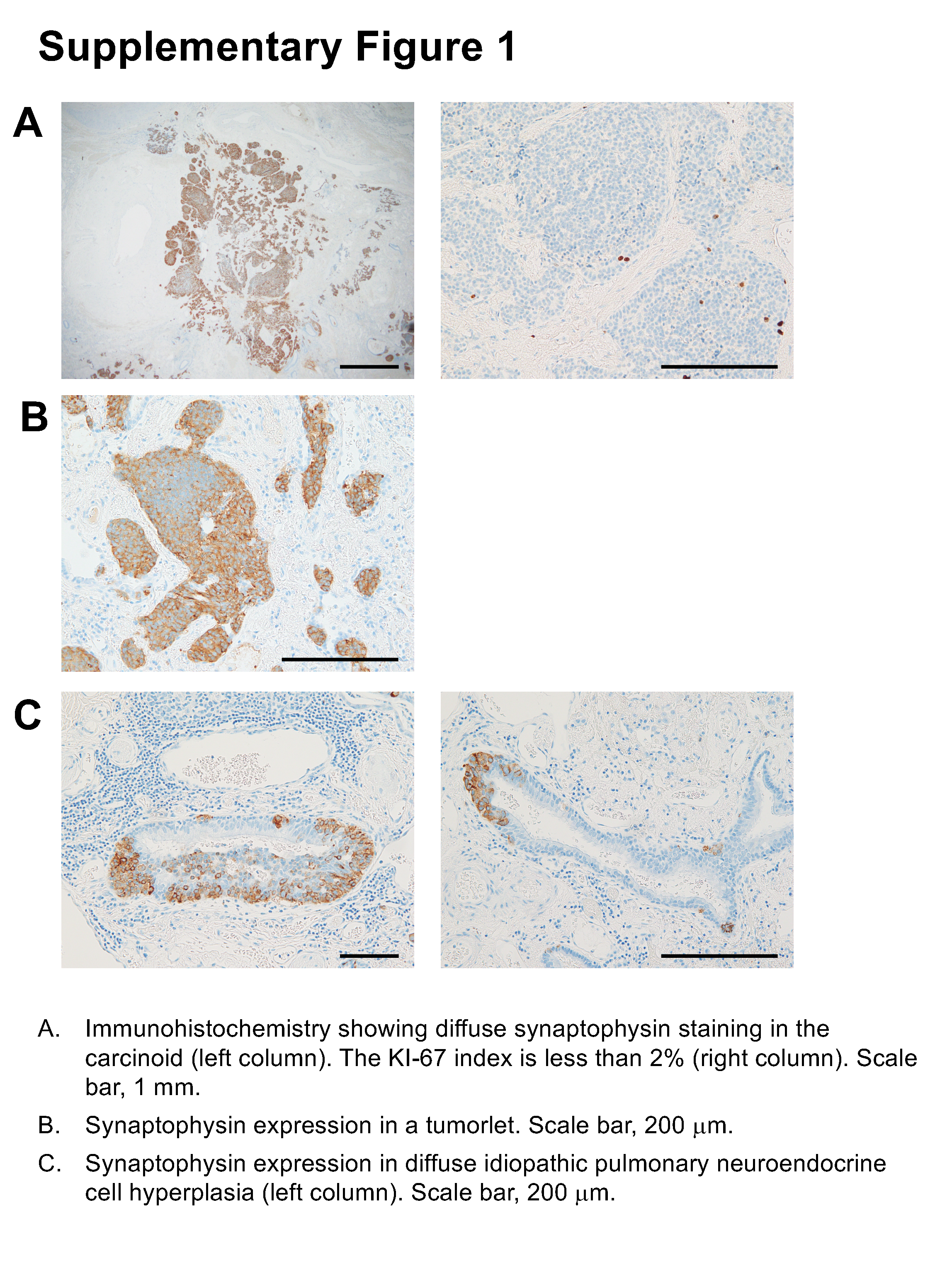

Supplement: Supplementary file 1 — Figure S1 (a) Immunohistochemistry showing diffuse synaptophysin staining in the carcinoid (left column). The KI‐67 index is less than 2% (right column). Scale bar, 1 mm. (b) Synaptophysin expression in a tumorlet. Scale bar, 200 μm. (c) Synaptophysin expression in diffuse idiopathic pulmonary neurorndocrine cell hyperplasia (left cloumn). Scale bar, 200 μm [file TCA-13-1227-s001.tif]
